# Influence of Redox Stress on Crosstalk between Fibroblasts and Keratinocytes

**DOI:** 10.3390/biology10121338

**Published:** 2021-12-16

**Authors:** Pradeep Bhartiya, Kai Masur, Debarati Shome, Neha Kaushik, Linh N. Nguyen, Nagendra Kumar Kaushik, Eun Ha Choi

**Affiliations:** 1 Plasma Bioscience Research Center, Applied Plasma Medicine Center, Department of Electrical and Biological Physics, Kwangwoon University, Seoul 01897, Korea; pradeepindian65@gmail.com (P.B.); nhatlinhusth@gmail.com (L.N.N.); 2 ZIK *Plasmatis*, Leibniz Institute for Plasma Science and Technology, 17489 Greifswald, Germany; kai.masur@inp-greifswald.de (K.M.); debarati1992@gmail.com (D.S.); 3 Department of Biotechnology, College of Engineering, University of Suwon, Hwaseong 18323, Korea; neha.bioplasma@gmail.com

**Keywords:** skin homeostasis, physical plasma, cell migration

## Abstract

**Simple Summary:**

There has been significant scientific progress in skin care and skin damage repair, but the complete understanding of skin homeostasis is still beyond our reach. With an increase in environmental stress factors, the incidence rates of skin cancer and skin disorders are on the rise. Taken together with the incidence of scar- and burn-related morbidities, there is an urgent need to understand interactions between skin cells to develop novel therapies for the regeneration of healthy skin. One of the recurrent stress factors affecting the skin are the harmful free radicals, also referred to as oxidative stress. This study aimed to address the influence of oxidative stress on the interaction between two types of skin cells, keratinocytes and fibroblasts. The study utilized cold atmospheric plasma (CAP) to induce oxidative stress in cells and to assess the interactions between the two cell types. We showed that CAP can stimulate cells to enhance their proliferation and migration. This study provides a further understanding of skin cell regulation under stress conditions. Such knowledge may help in designing treatment therapies for rapid wound healing and skin repair.

**Abstract:**

Although the skin is constantly subjected to endogenous and exogenous stress, it maintains a homeostatic state through wound repair and regeneration pathways. Treatment for skin diseases and injury requires a significant understanding of the various mechanisms and interactions that occur within skin cells. Keratinocytes and fibroblasts interact with each other and act as key players in the repair process. Although fibroblasts and keratinocytes are widely studied in wound healing and skin remodeling under different conditions, the influence of redox stress on keratinocyte-fibroblast crosstalk has not been thoroughly investigated. In this study, we used cold atmospheric plasma (CAP) to generate and deliver oxidative stress to keratinocytes and fibroblasts and to assess its impact on their interactions. To this end, we used a well-established in vitro 3D co-culture model imitating a realistic scenario. Our study shows that low CAP exposure is biocompatible and does not affect the viability or energetics of fibroblasts and keratinocytes. Exposure to low doses of CAP enhanced the proliferation rate of cells and stimulated the expression of key genes (*KGF*, *MMP2*, *GMCSF*, *IL-6*, and *IL-8*) in fibroblasts, indicating the activation and initiation of the skin repair process. Additionally, enhanced migration was observed under co-culture conditions under the given redox stress conditions, and expression of the upstream regulator and the effectors of the Hippo pathway (YAP and CYR61, respectively), which are associated with enhanced migration, were elevated. Overall, this study reinforces the application of CAP and redox stress in skin repair physiology.

## 1. Introduction

Human skin, the largest organ of the human body, is involved in the regulation of body temperature and metabolic functions and acts as a barrier against harsh external environment, preventing skin damage [1,2,3]. The skin is a complex organ comprising an outer layer called the epidermis, which consists of keratinocytes, melanocytes, Merkel cells, and Langerhans cells, and an inner layer called the dermis, which is structurally more complex and consists of fibroblasts, sweat glands, lymphatic vessels, vasculature, and nerves [4,5,6,7]. These cells interact and coordinate a plethora of molecular changes to repair the skin when it is injured or aged. The growth, differentiation, and regeneration of skin require temporal and spatial molecular interactions between various cells, particularly keratinocytes and fibroblasts. Growing evidence indicates the existence of cellular crosstalk and double paracrine interactions between keratinocytes and fibroblasts [8,9,10]. During skin repair, fibroblast migration is enhanced, followed by the production and secretion of growth factors, such as keratinocyte growth factor (KGF), basic fibroblast growth factor (bFGF), insulin-like growth factor, and vascular endothelial growth factor A (VEGF-A). Activated fibroblasts synthesize growth factors and extracellular matrix (ECM) proteins that act on neighboring keratinocytes. Then, keratinocytes recruit, stimulate, and guide functions of multiple cell types, especially fibroblasts. When keratinocytes release interleukins (IL-1), proliferation and migration are enhanced in an autocrine manner or paracrine manner when KGF and ECM production in fibroblasts is upregulated [10]. The outcomes of these interactions are largely dependent on the proportions of key molecules, such as IL-1 and KGF, in the microenvironment. The crosstalk between fibroblasts and keratinocytes is key for tissue homeostasis and cutaneous wound healing and, hence, requires further investigation.

Cold atmospheric plasma (CAP) technology, which involves the generation of an ionized gas at room temperature and atmospheric pressure using electric discharges, is being widely studied in dermatology, as it is a noninvasive treatment that can have beneficial effects on skin decontamination and wound healing [11,12,13,14]. Previous studies have demonstrated the potential of CAP to accelerate skin repair [15]. In addition, several clinical studies on the treatment of chronic ulcers and pressure-induced ulcers in human patients have shown positive results [16,17]. A significant improvement in wound healing has also been reported using a skin graft donor [18]. Similarly, studies have shown that CAP can enhance wound healing in acute wounds [19]. Furthermore, Arndt et al. showed that CAP treatment for two minutes is sufficient to activate wound healing by inducing the expression of relevant molecules such as IL-6, IL-8, transforming growth factor B (TGF-b), collagen 1, and alpha-smooth muscle actin (α-SMA). They also reported enhanced migration, which is critical for skin repair [20]. Enhanced migration during wound healing was recently linked to the Hippo signaling cascade via Yes-associated protein (YAP) [21]. The positive effects of CAP are mostly attributed to low-dose treatment, which results in non thermal effects and the generation of sufficient amounts of reactive species, such as oxygen (ROS) and nitrogen (RNS), jointly named RONS. Oxidative stress can influence the expression of *CYR61* and *CTGF* in fibroblasts, which are target genes of YAP [22,23,24], a key regulator involved in redox homeostasis that can activate genetic programs useful for wound healing [25,26]. Altogether, these studies highlight the critical role of redox-induced signaling in skin homeostasis. Further comprehensive studies in this area are required to explore and understand this complex network.

This research focuses on the crosstalk of cells involved in wound healing and repair, as well as a novel therapy using the application of cold plasma. Further progress in understanding of skin lesions and scratch repair may assist in decoding the pathogenesis of skin conditions such as psoriasis, atopic dermatitis, etc. To fill the knowledge gap, the present work aims to investigate the reciprocal regulation of fibroblasts and keratinocytes with a focus on proliferation and migration under CAP treatment conditions. We evaluated the interactions between human dermal fibroblasts (GM00637) and epidermal keratinocytes using soft jet plasma and demonstrated the influence of redox stress on the activation of fibroblast gene expression required for wound healing and enhanced migration under co-culture conditions. The results of this study further reinforce the use of CAP for skin regeneration and repair, offering a new perspective for the treatment of chronic wounds.

## 2. Materials and Methods

### 2.1. Cell Culture

**Figure 1:**
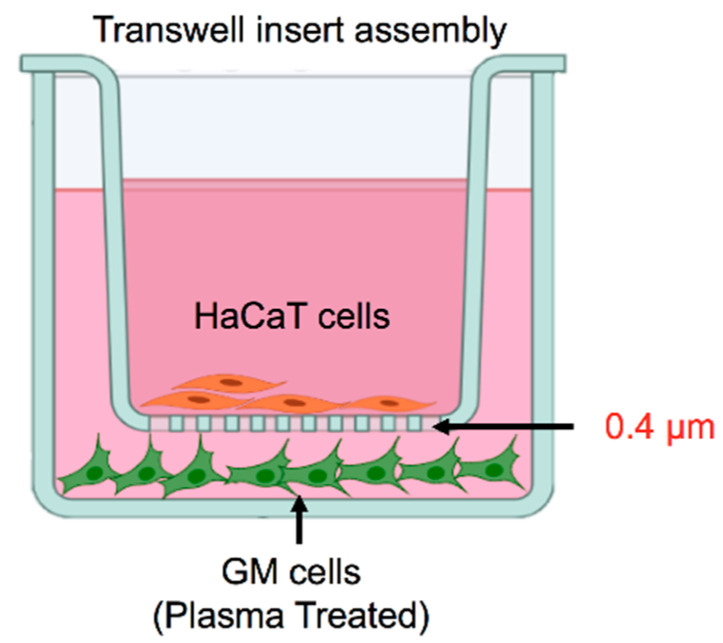
In vitro 3D co-culture model using fibroblasts (GM) at the bottom part of well and Keratinocytes (HaCaT) on the upper layer inside the insert.

The skin-derived fibroblast cell line GM00637 (GM) purchased from the Coriell Institute, USA, and skin keratinocytes (HaCaT) obtained from the Yonsei University, Korea, were cultured in RPMI 1640 (Welgene, Gyeongsan, Korea) medium supplemented with 10% fetal bovine serum (FBS, RDTech, USA) and 1% penicillin/streptomycin. For scratch assays, cells were seeded either in mono- or co-culture conditions, according to experimental requirements and were incubated in an Image Lock culture 96-well plate (Essen BioScience, Ann Arbor, MI, USA) in medium with 2% FBS for 15–18  h before the start of the experiment. The co-culture setup for the study of the crosstalk is illustrated in Figure 1.

### 2.2. Plasma Exposure

**Figure 2:**
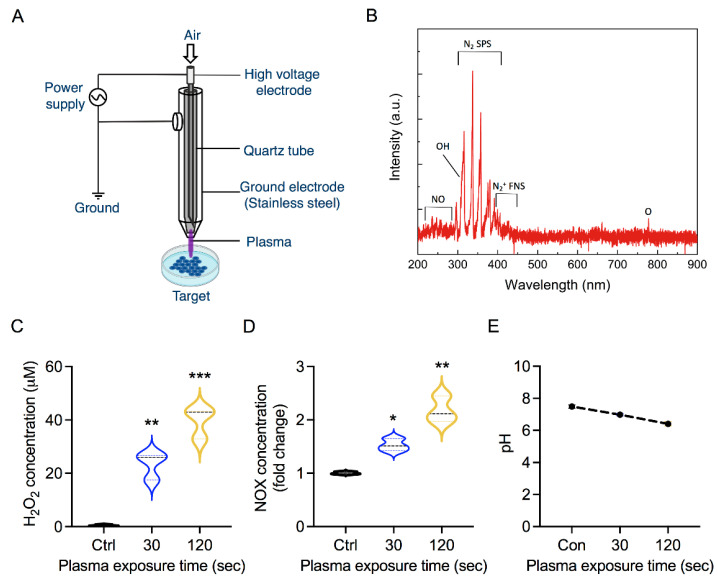
Physical characterization of soft jet plasma. (**A**) Schematic representation of soft jet plasma, (**B**) optical emission spectrum of the jet plasma, (**C**) H_2_O_2_ concentration in medium, (**D**) relative NO_x_ concentration in medium, and (**E**) pH changes in medium. Statistical significance determined by two-tailed Student’s *t*-test with 95% confidence interval is represented as * *p* < 0.05, ** *p* < 0.01, and *** *p* < 0.001.

CAP was generated with the help of a simple coaxial plasma jet using ambient air, as shown in Figure 2A [27]. The construction and the electrical and physical characterization of the plasma jet have been explained systematically in our previous study [27]. Briefly, the plasma jet was powered by applying an AC voltage of 2.2 kV and a frequency of 42 kHz. The operation duty cycle was fixed at 7% time-on/time-off. The gas flow rate used was set at 1 LPM (liter per minute). The GM cells were cultured in 2  mL of RPMI 1640 supplemented with 2% FBS in a 6-well plate and subjected to plasma treatment for 30 s and 120 s. For the scratch wound assay, 2 mL of CAP-treated media was immediately used to treat the cells. Cells incubated with RPMI medium without plasma exposure served as controls for all experiments.

### 2.3. Cellular Metabolic Viability

To assess metabolic activity, HaCaT cells (in monoculture) and GM cells (in monoculture) were seeded at a density of 1 × 10^4^ in a 96-well plate (SPL, Pocheon-si, Korea) one day before the start of the experiment. For the co-culture condition, GM and HaCaT cells were seeded at density of 0.3 × 10^4^ and 0.6 × 10^4^, respectively. After 24 h incubation with CAP-activated media, alamarBlue dye (Thermofisher, Waltham, MA, USA) was added to detect metabolically active cells. The plate was incubated for 2 h at 37 °C, and fluorescence was measured using a plate reader (BioTek, Winooski, VT, USA) at 540 nm and 600 nm. For comparison, the fluorescence intensity of untreated control samples was set to 100% metabolic activity. All further fluorescent values obtained in CAP-treated groups were normalized to those of untreated controls [28,29].

### 2.4. Measurement of Cellular ATP

Cellular ATP was estimated according to the manufacturer’s protocol using the Cell Titer-Glo Assay (Promega, Madison, WI, USA). To this end, we seeded 5 × 10^3^ cells per well in a 96-well plate with a volume of 100 µL of cell culture medium in monoculture and co-culture conditions. After 24 h of incubation, an equal volume of prewarmed reagent was added to the cells, followed by incubation for 1 h at 37 °C. After 1 h of incubation, luminescence was measured using a microplate reader (BioTek, Winooski, VT, USA) [30].

### 2.5. Mitochondria Membrane Potential Analysis

Mitoflow reagent (Cell Technology, Fremont, CA, USA) was used to determine mitochondrial integrity by measuring the changes in DeltaPsi. After harvesting, the cells were stained with Mitoflow reagent according to the manufacturer’s protocol. Samples were then analyzed using the BD FACSVerse system and FACS suite software.

### 2.6. Cell Migration Assay

The cell migration activity of HaCaT and GM cells was assessed using the Essen Bioscience wound assay. To this end, co-cultures of HaCaT and GM cells were used. For co-culture, 2 × 10^4^ GM/well were cultured in image lock plates and incubated for 3–4 h until the fibroblasts attached to the plates, and then, 4 × 10^4^ HaCaT cells/well were seeded on the top of the fibroblasts at a ratio of 1: 2 fibroblasts: keratinocytes. After 16–18 h, the cells were starved using 2% FBS for 16–18  h. We used an Essen BioScience wound maker to create scratches, and the cells were then incubated with a plasma-treated medium (30 s or 120 s) as treatment groups or untreated medium as the control groups. The rate of cell migration was measured using an IncuCyte live cell analysis system for up to 48 h. The relative wound density (%), defined as the ratio of the cell-occupied area of the initial wound area to the total wound area, was quantified using the IncuCyte 2019 B Software.

### 2.7. Quantitative PCR (qPCR) Analysis

RNA from plasma-treated fibroblast cells and HaCaT cells cultured in monoculture and co-cultured conditions was extracted using RNAiso Plus (Takara, Kusatsu, Japan) following the RNA extraction method (Invitrogen). Total RNA (2 µg) was used to synthesize template cDNA using a reverse transcription kit (Enzynomics) consisting of MMLV reverse transcriptase, RNAse inhibitor, and MMLV RT buffer with dNTPs on a thermocycler (Applied Biosystems, Waltham, MA, USA) following the manufacturer’s protocol. For quantification, qPCR reactions were prepared with iQ SYBR Green Supermix (Bio-Rad) and carried out on an iCycler IQ real-time system (Bio-Rad). The primer sequences used in this study are given in Table 1 below.

### 2.8. Statistics

The results are expressed as the mean ± SD of triplicate experiments from three independent experiments. Student’s *t*-test was used to analyze significant differences between groups, whereas one-way analysis of variance was used for comparison between multiple groups. Significance was considered as * *p* < 0.05, ** *p* < 0.01, and *** *p* < 0.001. 

## 3. Results

### 3.1. Soft Jet Plasma and RONS Concentration 

Taking advantage of the ambient environment, CAP can generate RONS in a liquid target through plasma–liquid interactions [11]. Figure 2A shows an illustration of a plasma device used for the generation of RONS conferring oxidative stress in samples. The existence of the RONS in the plasma can be confirmed through the optical emission spectroscopy (OES). Thus, we recorded the OES spectrum of the CAP using an Ocean optic HR4000 spectrometer to identify the primal plasma phase RONS (Figure 2B). For the reactive nitrogen species, the emission bands of nitric oxide (NO), Nitrogen second positive system (N_2_ SPS), and Nitrogen first negative system (N_2_+ FNS) were detected at the ranges of 200–250 nm, 310–400 nm, and 400–450 nm, respectively. The OES spectrum also confirms the existence of some reactive oxygen species such as OH radical at 309 nm and atomic oxygen at 777 nm. The impact of plasma treatment on a biological sample depends on the plasma operation parameters, which dictate the amount of RONS generated, as shown in Figure 2A,B. To this end, we measured the concentration of RONS produced by the soft jet plasma in our experiment. As shown in Figure 2C,D, CAP exposure led to dose-dependent increases in H_2_O_2_ and NO_x_ levels, respectively. The H_2_O_2_ concentration was measured to be approximately 20 µM after 30 s and increased up to 60 µM after 120 s of plasma treatment. Simultaneously, the plasma-generated species could affect the pH of solution to trigger metabolic changes or cell death pathways in cells. We observed a decrease in the pH of the media with increasing CAP exposure time (Figure 2E). After treatment times of 30 s and 120 s, the pH of the media dropped from 7.4 to 6.9 and 6.3, respectively. The results confirmed that the pH value was affected by the plasma treatment but was still within the biocompatible range.

### 3.2. GM and HaCaT Cell Proliferation Is Enhanced after CAP Treatment

**Figure 3:**
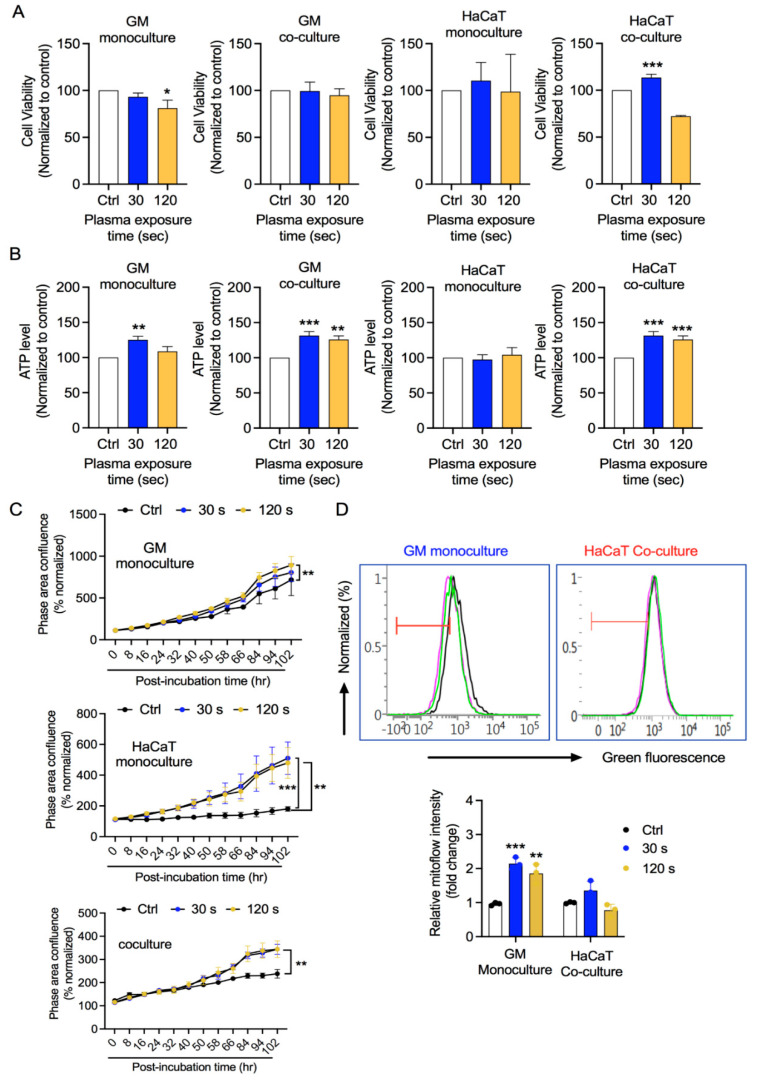
Effect of CAP exposure on metabolic activity and cell growth. (**A**) Metabolic activity of GM and HaCaT cells after CAP exposure, (**B**) relative energy (ATP) levels in cells after CAP exposure, (**C**) representative graphs indicating cell proliferation rate measured up to 5 days using Incucyte suite, and (**D**) normalized spectra and graph of mitoflow intensity in GM and HaCaT cells after CAP exposure as indicated. Statistical significance determined using two-tailed Student’s *t*-test is denoted as * *p* < 0.05, ** *p* < 0.01, and *** *p* < 0.001.

We performed a preliminary assessment of CAP treatment effect on fibroblasts (GM) and keratinocytes (HaCaT). To this end, we cultured fibroblasts and keratinocytes separately in monoculture conditions and together in co-culture conditions. In co-culture, GM cells were seeded inside 6-well culture plates and HaCaT cells were seeded onto an insert placed above GM cells, allowing fluid exchange between the two cell types. As the cells reached 70–80% confluency, the complete medium was replaced with 2% FBS medium for 16–18 h. The pre-starved HaCaT cells were temporarily displaced to allow CAP exposure to the pre-starved GM cells for the indicated times. The pre-starved HaCaT cells were again placed above the GM cells, and then, the plate was incubated. After 2 days, the cells were harvested and changes in metabolic activity and the ATP levels were assessed. As shown in Figure 3A, a low CAP exposure did not reduce the metabolic activity of GM cells either in monoculture or co-culture conditions. High-dose CAP exposure led to slight growth inhibition of GM cells in monoculture, which was recovered in coculture conditions. HaCaT cells showed reduced metabolic activity under high CAP exposure under co-culture conditions, while metabolic activity remained unaffected in monoculture conditions in both low or high CAP exposure. Another way of assessing cellular metabolic activity is through cellular energetics. We measured ATP levels in cells after CAP exposure. As shown in Figure 3B, GM cells undergo increases in ATP levels in both monoculture and coculture conditions after CAP exposure. HaCaT cells showed enhanced ATP levels in coculture conditions, while no significant changes were seen in monoculture conditions. Furthermore, we checked the proliferation rates of GM and HaCaT cells using the software Incucyte 2019 B suite. CAP exposure enhanced the proliferation rates of GM and HaCaT cells under both conditions (Figure 3C). Metabolic activity and ATP levels of cells are positively associated with mitochondrial health [31,32,33,34]. From the results of metabolic activity, ATP, and proliferation, we evaluated the mitochondrial membrane potential (DeltaPsi) in the GM cells in monoculture conditions and HaCaT from coculture conditions after inducing redox stress. CAP exposure led to an increase in mitoflow intensity in GM cells, while there were no significant alterations in mitochondrial membrane potential in keratinocytes compared with their respective control groups (Figure 3D). Taken together, CAP exposure did not alter the cellular metabolic activity and energetics of either fibroblasts or keratinocytes at detrimental levels. CAP exposure enhanced the proliferation of cells.

### 3.3. Low-Dose Plasma Enhances the Expression of Crosstalk Molecules between Fibroblasts and Keratinocytes

**Figure 4:**
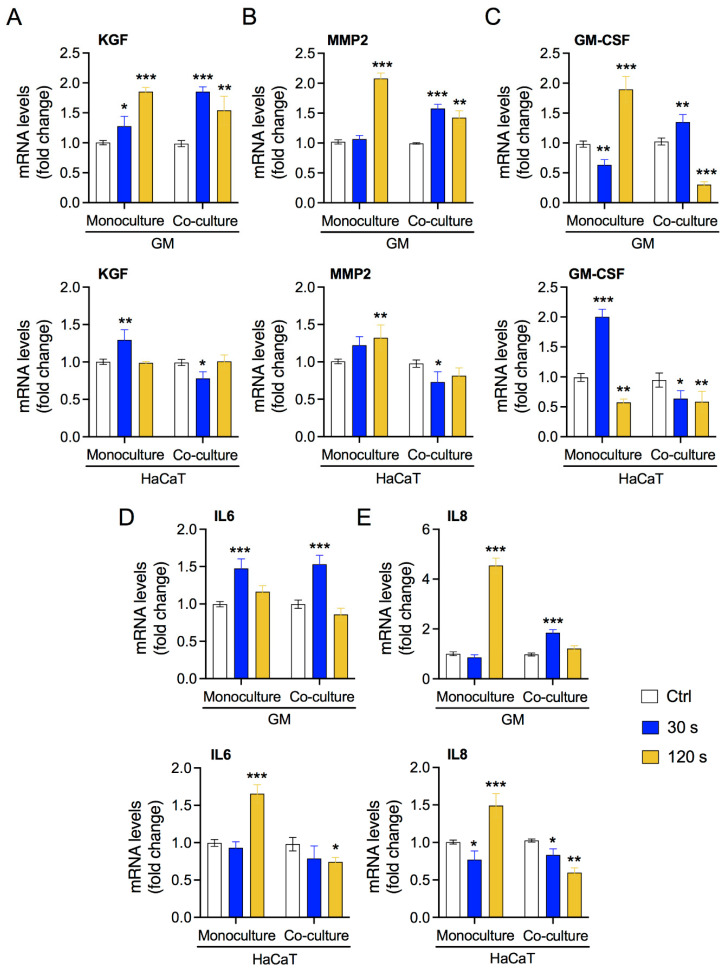
Influence of CAP exposure on gene expression levels in GM and HaCaT cells. Gene expression profile quantified using qPCR for (**A**) KGF, (**B**) MMP2, (**C**) GM-CSF, (**D**) IL-6, and (**E**) IL-8. The top horizontal panel represents gene expression in GM cells, and the bottom panel represents expression in HaCaT cells. Statistical significance determined using two-tailed Student’s *t*-test is denoted as * *p* < 0.05, ** *p* < 0.01, and *** *p* < 0.001.

Several studies have reported the interaction between fibroblasts and keratinocytes [9]. In damaged tissue, keratinocytes stimulate fibroblasts to upregulate key molecules, such as KGF, cytokines, and components of the ECM. Activated fibroblasts promote the proliferation and migration of keratinocytes, completing paracrine crosstalk. We assessed the expression levels of the marker genes required for these interactions in our system. As shown in Figure 4A,B, CAP exposure induced the expression of KGF and MMP2 in both monoculture and co-culture conditions mainly in fibroblasts GM, as expected. We also assessed the expression of these genes in keratinocytes HaCaT and observed significant increase in monoculture condition but unaffected or reduced in coculture conditions, indicating fibroblast-specific expression. Additionally, CAP exposure induced expression of key cytokines in our in vitro system (Figure 4C–E). GM cells showed consistent elevation of GM-CSF, IL-6, and IL-8 expression by low CAP exposure under monoculture and coculture conditions, while it was reduced or unaffected by high CAP exposure. In HaCaT cells, low CAP exposure enhanced the of GM-CSF but failed to increase IL-6 and IL-8 expression under monoculture conditions. On the contrary, high CAP exposure reduced GM-CSF expression but elevated IL-6 and IL-8 expression under monoculture conditions. No increase in the expression levels of cytokines was observed in HaCaT under coculture conditions. These results were further validated with ROS scavenger—N-acetyl-L-cysteine (NAC) in coculture conditions. NAC pretreatment with CAP exposed groups showed reduced levels of cytokines in both GM and HaCaT cells, whereas GM-CSF in HaCaT cells was an exception (Supplementary Figure S1). This indicates that the induction of cytokines could be through CAP-generated ROS. Overall, these results indicate that CAP exposure can lead to the activation of fibroblasts by modulating the gene expression of essential molecules.

### 3.4. Fibroblast and HaCaT Cell Migration Is Enhanced under Low-Plasma Conditions

**Figure 5:**
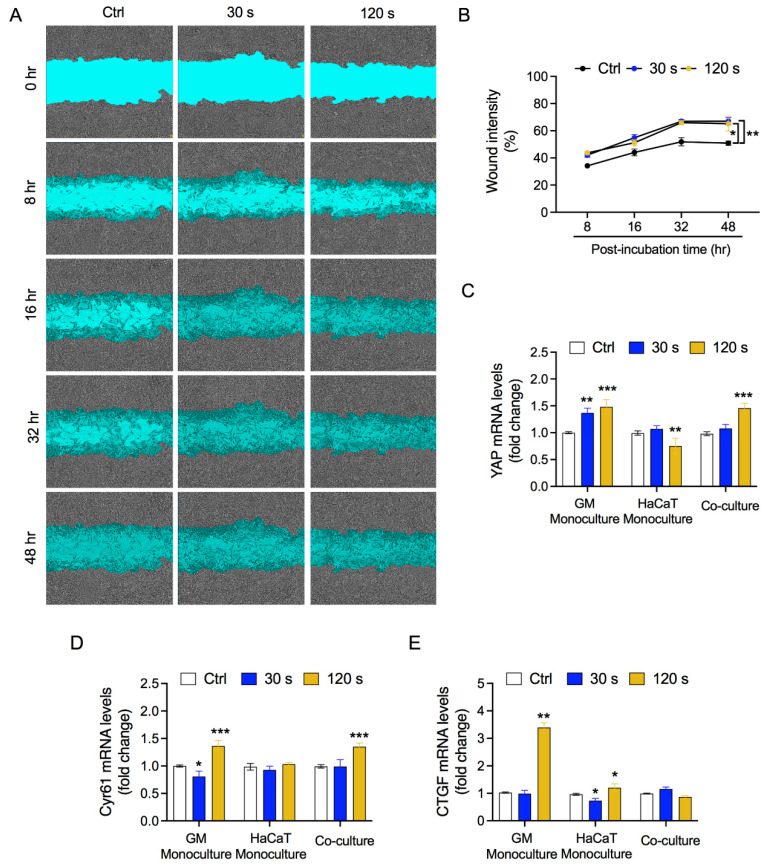
CAP exposure enhances cell migration rate in co-culture conditions. (**A**) Cell migration assay in co-culture conditions upon CAP exposure (30 s and 120 s). (**B**) Graph indicating wound density. Gene expression profiles of (**C**) YAP, (**D**) CYR61, and (**E**) CTGF were measured using qPCR. Statistical significance determined by two-tailed Student’s *t*-test is denoted as * *p* < 0.05, ** *p* < 0.01, and *** *p* < 0.001.

Enhanced proliferation and migration of both fibroblasts and keratinocytes are required for aiding the wound healing process. Keratinocytes and fibroblast can regulate their proliferation through paracrine interactions *in vitro* coculture systems involving trans-well or inserts. However, their migration is hindered in the absence of direct contact between the two cell types. To further evaluate the migration of cells after CAP treatment, we cultured GM and HaCaT cells together in Incucyte Bioessen 96-well plates at high density to form a monolayer. A Sartorius wound maker was used to create uniform wounds. The cells were treated with CAP-exposed medium and kept under continuous observation using Incucyte suite, which was used to capture and analyze the wound density in the control and treatment groups. The wound density in the CAP-treated wells was higher than that in the control wells (Figure 5A). The migration rate was enhanced after 16 h of CAP exposure and remained consistently high until 48 h (Figure 5B). As the migration of fibroblasts and keratinocytes has been linked to the activity of the Hippo signaling pathway, we assessed the expression levels of the key ligands that regulate the Hippo cascade using RT-qPCR. As shown in Figure 5C, YAP mRNA expression was elevated in GM cells in monoculture, indicating a direct effect of CAP exposure. Elevation in YAP expression was sustained through high CAP exposure under coculture conditions. In HaCaT cells, YAP levels remained unaffected by low CAP exposure but was reduced by high CAP exposure under monoculture conditions. We also determined the levels of CYR61 and CTGF, which help in the crosstalk between fibroblasts and keratinocytes. As shown in Figure 5D, CAP exposure induced the CYR61 mRNA expression in GM cells under monoculture conditions and maintained high levels in coculture conditions. HaCaT cells showed no significant difference in CYR61 mRNA expression after CAP exposure. Figure 5E indicates the mRNA expression levels of CTGF. CAP exposure induced the expression of CTGF in GM cells under monoculture conditions. However, this elevation was not observed under co-culture conditions. CTGF mRNA expression remained unaffected in HaCaT cells after CAP exposure. Thus, CAP exposure can enhance the migration of fibroblasts and keratinocytes in coculture conditions through upregulation of the Hippo signaling axis.

## 4. Discussion

The regulation of skin homeostasis requires the participation of keratinocytes and fibroblasts and the establishment of delicate interactions between these two cell types. Any injury makes these cells metabolically more active, facilitating wound healing or preventing skin fibrotic disorders. Accurately replicating keratinocyte–fibroblast interactions *in vitro* has been challenging, highlighting the need for new skin models to understand skin homeostasis. The most simplistic approach to assessing fibroblasts–keratinocytes interactions involves the use of a conditioned medium of one cell type to be transferred to another. This method, though quite useful and straightforward, has limitations owing to the differential media requirements of the cell types under investigation [35,36,37,38]. Various research groups have used physical co-cultures of fibroblasts and keratinocytes utilizing a trans-well system wherein keratinocytes are cultured in the upper well representing the epidermal layer and fibroblasts in the bottom well to represent the dermal layer [39,40,41,42]. In our study, we employed a trans-well system to assess keratinocyte-fibroblast crosstalk under redox stress conditions (Figure 1). This model helps to investigate the influence of fibroblasts on keratinocytes under oxidative stress conditions in the absence of secondary influences generated in the skin. We used CAP to generate redox stress mediated by a cocktail of reactive oxygen and nitrogen species, including short-lived free radicals and relatively stable H_2_O_2_. Our plasma jet generated significant levels of H_2_O_2_ and NO_x_ in the medium, even at low exposure doses (Figure 2). CAP has previously been reported to generate significant levels of RONS in biological liquids, including deionized water, phosphate-buffered saline, and growth medium [43]. The generation of active reactive species may take place through plasma–air interface reactions or plasma–liquid interface reactions. This allows for both direct (plasma source) and indirect use (plasma activated liquids) of plasma. CAP has been studied in skin biology owing to its biocompatibility (Figure 2) [44,45,46]. Further assessment showed that redox stress did not reduce the viability and energy metabolism of fibroblasts or keratinocytes (Figure 3A,B). This was further supported by the insignificant alteration in the mitochondrial membrane potential (DeltaPsi). ATP synthesis and cellular health are largely dependent on the structural integrity and function of mitochondria. Exposure to low doses of oxidative stress can stimulate non-malignant cell proliferation, while high doses may inhibit cellular metabolism and eventually proliferation [47,48,49,50,51]. This was expected as non-malignant cells are assumed to have a high tolerance towards redox stress with a low death rate compared with malignant cells. Overall, CAP stimulated the cells to proliferate under co-culture conditions (Figure 3D), as seen in previous reports including wound healing studies [21,48,52]. These studies stress the differential responses of cells depending on the exposure parameters of CAP or ROS levels. Low doses of plasma can activate the NF-kB cascade and cyclinD1 expression leading to cell proliferation.

Skin injury induces several changes in fibroblasts and keratinocytes. Upon injury, the transcription of ECM genes and several other proteins is activated in fibroblasts [53,54,55,56]. In line with previous studies indicating activation of fibroblasts for wound healing, we observed upregulation of several genes, such as *KGF*, *MMP-9*, and interleukins (Figure 4) [10,42]. Upregulation of these genes has been attributed to the presence of keratinocytes in co-culture systems [57,58]. Most studies have shown keratinocyte-dependent ECM production and signaling regulation in fibroblasts, but studies on the reciprocal crosstalk studies have been scarce [38,42,59,60,61]. The study of fibroblast-dependent changes in keratinocytes has primarily focused on keratin production and keratinocyte proliferation [62,63,64]. In our study, we assessed the expression levels of these genes in keratinocytes to determine the influence of oxidative stress-activated fibroblasts on keratinocytes. Keratinocytes showed changes in the interleukin profile, which is compatible with partially or completely differentiated keratinocytes, as they synthesize and regulate a distinct class of proteins that may react differentially with exogenous cues. As expected, keratinocytes showed no significant alterations in the expression of *KGF* and *GMCSF*. Keratinocytes express KGF and GM-CSF receptors via autocrine regulation mediated by TGF-alpha or paracrine regulation mediated by KGF and GM-CSF secreted by fibroblasts [64]. While GM-CSF is enhanced at low CAP, it drops at high CAP conditions—and when stress is elevated at high CAP, the IL-6 and IL-8 concentrations raise. A pretty good example for/ of oxidative stress is that a well-balanced plasma treatment is necessary to orchestrate the cells in co-culture or even a real wound. This induction in the expression of cytokines was primarily due to ROS generated by CAP (Supplementary Figure S1). Activated fibroblasts and keratinocytes stimulate each other to proliferate and migrate in injured skin or in equivalent models. In our *in vitro* co-culture system, we observed increased migration in line with previous reports (Figure 5) [37,65,66]. The increased migration of keratinocytes could be due to KGF-induced production of IL-19 by HaCaT cells, while soluble factors present in a HaCaT-conditioned medium might have influenced fibroblast migration [37,65]. A recent report suggested that the impact of redox stress affects the modulation of soluble factors in a conditioned medium, leading to increased migration [21]. This study also linked the redox stress to the Hippo cascade, which are important for wound healing. Our study showed elevated levels of YAP and CYR61, which are key upstream regulators of the Hippo signaling pathway, consistent with a previous report [21]. Taken together, these results indicate that redox stress induces Hippo-or Hippo-like specific pathways for wound healing.

The clinical importance of skin homeostasis is evidenced by the association between cutaneous defects and morbidity. The increasing number of skin-related issues has become a daunting issue that requires novel therapies and research. Current therapy modules and research models are limited to skin grafts for wounds and burns. Additional research using novel biocompatible options, such as CAP, is necessary to use new biocompatible options. In recent years, many clinical studies have investigated the effects of CAP, especially in wound healing and melanomas, with the aim of understanding interactive networks in skin biology. Few studies have been conducted to investigate the role of CAP, ROS, and associated electrical signals in the proliferation and differentiation of epidermal cells, fibroblasts, and myofibroblast transdifferentiation [67,68]. However, redox-induced signaling mechanisms are poorly understood and require further research. The establishment of redox stress *ex vivo* complementing 3D *in vitro* models such as those used in this study could overcome its limitations and recapitulate the skin repair process. Alternatively, extensive transcriptome analysis of cells derived from healthy skin and oxidative stress damaged skin will scale up our knowledge. Taken together, new approaches with CAP will help in deciphering the subtle mechanisms involved in skin repair.

## 5. Conclusions

In conclusion, CAP exposure can engage with keratinocyte-fibroblast crosstalk at the onset of injury and can modulate it further to enhance the repair process. To this end, CAP may stimulate the cells to proliferate and upregulate redox-induced pathways such as the Hippo signaling cascade, which may facilitate paracrine signaling between cells. Although CAP-mediated biological responses are largely attributed to RONS, additional research is required to understand the synergistic and additive functions of CAP, especially in the skin.

## Figures and Tables

**Table 1:** List of primer sequences used in this study.

Gene	L/R	Primer Sequence	Product (bp)
Actin	L	5’ GGC ATC CTC ACC CTG AAG TA 3’	82
	R	5’ AGG TGT GGT GCC AGA TTT TC 3’	
KGF	L	5′ CAC CCG GAG CAC TAC ACT AT 3′	185
	R	5′ CTT CTT GTG TGT CGC TCA GG 3′	
MMP2	L	5′ CTA CTG AGT GGC CGT GTT TG 3′	174
	R	5′ TCC CTG AGG TTC TCT TGC TG 3′	
GMCSF	L	5′ CAT GTG TGG CTG ATA AGG GC 3′	166
	R	5′ GCC ACA TCC TCC AGA GAA CT 3′	
IL-6	L	5’ TTC TTG GGA CTG ATG CTG 3’	180
	R	5’ CTG GCT TTG TCT TTC TTG TT 3’	
IL-8	L	5’ CAG GAA TTG AAT GGG TTT GC 3’	180
	R	5’ AAA CCA AGG CAC AGT GGA AC 3’	
YAP	L	5′ TGT CCC AGA TGA ACG TCA CA 3′	191
	R	5′ GTT CAT GGC AAA ACG AGG GT 3′	
CYR61	L	5′ GTG TGA AGA AAT ACC GGC CC 3′	199
	R	5′ CTG TAG AAG GGA AAC GCT GC 3′	

## Data Availability

Not applicable.

## Supplementary Materials

The following are available online at https://www.mdpi.com/article/10.3390/biology10121338/s1. Figure S1: N-acetyl-L-cysteine inhibits CAP induced cytokines. The top horizontal panel shows gene expression profile for (A) GM-CSF and (B) IL-8 in GM cells, whereas bottom horizontal panel represents gene expression profile for (C) GM-CSF and (D) IL-8 in HaCaT cells. Statistical significance was determined using two-tailed Student’s *t*-test and is denoted as * *p* < 0.05, ** *p* < 0.01, and *** *p* < 0.001.
