# Supplementary material for: Influence of Redox Stress on Crosstalk between Fibroblasts and Keratinocytes
Source: Biology (Basel). 2021 Dec 16;10(12):1338. doi: 10.3390/biology10121338 (PMC8698713; doi:10.3390/biology10121338)
Supplement: Supplementary file 1 [file biology-10-01338-s001.zip › biology-1446722-supplementary.pdf]

Supplementary Information

Influence of redox stress on crosstalk between fibroblasts and keratinocytes

Pradeep Bhartiya[1], Kai Masur[2], Debarati Shome[2], Neha Kaushik[3], Linh N. Nguyen[1], Nagendra Kumar Kaushik[1*], Eun Ha Choi[1*]

[1] Plasma Bioscience Research Center, Applied Plasma Medicine Center, Department of Electrical and Biological Physics, Kwangwoon University, 01897, Seoul, Korea; pradeepindian65@gmail.com (P.B.), nhatlinhusth@gmail.com (L.N.N.)

[2] ZIK *plasmatis*, Leibniz Institute for Plasma Science and Technology, 17489 Greifswald, Germany; kai.masur@inp-greifswald.de (K.M.)

[3] Department of Biotechnology, College of Engineering, University of Suwon, Hwaseong, 18323, Korea; neha.bioplasma@gmail.com

* Correspondence: kaushik.nagendra@kw.ac.kr (N.K.K.) and ehchoi@kw.ac.kr (E.H.C.)

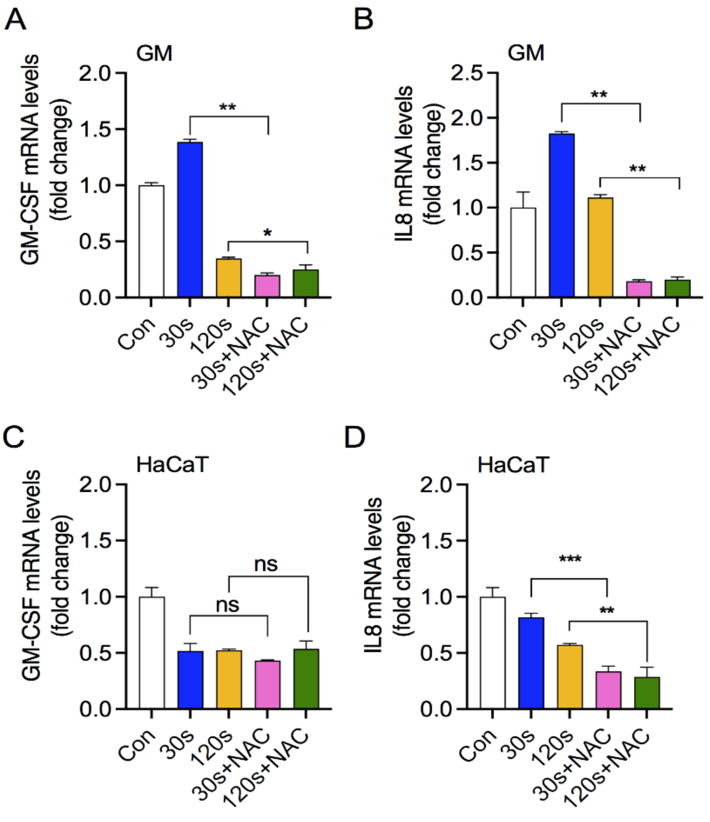

Figure S1: N-acetyl-L-cysteine inhibits CAP induced cytokines. The top horizontal panel shows gene expression profile for (A) GM-CSF and (B) IL-8 in GM cells, whereas bottom horizontal panel represents gene expression profile for (C) GM-CSF and (D) IL-8 in HaCaT cells. Statistical significance was determined using two-tailed Student's t-test and is denoted as * $p < 0.05$, ** $p < 0.01$, and *** $p < 0.001$
